# Case Report: Diagnostic pitfalls and laparoscopic repair of a delayed large right-sided traumatic diaphragmatic hernia

**DOI:** 10.3389/fsurg.2026.1956256

**Published:** 2026-09-22

**Authors:** Mengping Duan, Yan Qiao, Zhoubin Duan, Shihu Li, Jianxun Guan, Fating Dong, Xiaoyan Yin, Xiangyu Wang, Chungui Liu, Fujin Ye, Daihong Song

**Affiliations:** 1 Chuxiong Prefecture People's Hospital, Chuxiong, Yunnan, China; 2 Chuxiong Prefecture Hospital of Traditional Chinese Medicine, Chuxiong, Yunnan, China; 3 Department of Colorectal Surgery, Guangdong Institute of Gastroenterology and Guangdong Provincial Key Laboratory of Colorectal and Pelvic Floor Diseases, The Sixth Affiliated Hospital, Sun Yat-sen University, Guangzhou, Guangdong, China

**Keywords:** blunt trauma, bowel herniation, case report, delayed diagnosis, laparoscopy, right-sided diaphragmatic hernia, traumatic diaphragmatic hernia

## Abstract

**Background:**

Delayed right-sided traumatic diaphragmatic hernia (TDH) may be overlooked during polytrauma assessment because concomitant injuries dominate attention and the liver can obscure diaphragmatic discontinuity. Subsequent respiratory and gastrointestinal symptoms may remain nonspecific until obstruction or visceral compromise develops.

**Case description:**

A 50-year-old man presented with more than 20 h of nausea and vomiting 19 months after severe blunt trauma. Since the injury, he had experienced intermittent dyspnea, chest tightness, postprandial fullness, abdominal distension, and abdominal pain. His admission oxygen saturation was 94%, with no supplemental oxygen recorded. Examination revealed diminished right-sided breath sounds and bowel sounds in the lower right hemithorax. Computed tomography demonstrated discontinuity of the right hemidiaphragm and herniation of the stomach, liver, gallbladder, and hepatic flexure. Transabdominal laparoscopy confirmed an approximately 8.0 cm×15.0 cm posterolateral defect. After adhesiolysis, all herniated organs were reduced and remained viable. The defect was closed primarily without mesh because the mobilized edges were robust and could be approximated without a residual gap, visible tearing, or excessive tension. Respiratory and gastrointestinal symptoms resolved, and early postoperative imaging confirmed restoration of the abdominal viscera below the diaphragm.

**Conclusion:**

After high-energy trauma, discordant respiratory and meal-related symptoms plus intrathoracic bowel sounds should prompt targeted multiplanar CT review. In selected stable patients, transabdominal laparoscopy can combine reduction, assessment of visceral viability, and repair. The approach and need for reinforcement should be individualized according to adhesions, tissue quality, and achievable edge approximation.

## 1 Introduction

Traumatic diaphragmatic injury may be overlooked during initial resuscitation, particularly when neurologic, orthopedic, and pulmonary injuries dominate clinical attention (1, 2). A small rupture may remain clinically silent until progressive herniation causes obstruction, strangulation, perforation, or cardiorespiratory compromise (1). Right-sided injury is especially difficult to recognize because the liver can buttress the defect and obscure diaphragmatic discontinuity. On computed tomography (CT), both direct discontinuity and indirect signs warrant deliberate multiplanar review. These include the collar, dependent-viscera, and dangling-diaphragm signs (3, 4).

Published cases already establish that delayed right-sided traumatic diaphragmatic hernia (TDH) can mimic biliary disease, progress to bowel strangulation or perforation, and require different minimally invasive approaches (5–10). The clinically useful questions are how to recognize the condition before visceral compromise and how to select an operative approach when the liver and chronic adhesions limit access.

We describe a delayed large right-sided TDH presenting with acute obstructive gastrointestinal symptoms 19 months after blunt trauma. The report links a specific bedside clue to targeted CT interpretation and details an organ-preserving transabdominal laparoscopic repair. This case is reported in accordance with the CARE guideline (11).

## 2 Case description

### 2.1 Patient information

A 50-year-old man presented to the emergency department with nausea and vomiting for more than 20 h. Nineteen months earlier, he had sustained severe blunt trauma with intracranial hematoma, multiple cerebral contusions and lacerations, pelvic fracture, femoral fracture, and pulmonary contusion. The initial injuries required surgical intensive care and orthopedic surgery.

The original CT dataset and lung-window images from the trauma admission could not be retrieved for retrospective review. The pulmonary contusion is therefore reported from the contemporaneous medical record rather than from re-interpretation of the original images.

During the interval after trauma, he experienced intermittent dyspnea, chest tightness, postprandial fullness, abdominal distension, and abdominal pain. He had not undergone systematic reassessment for these symptoms. The episode of care is summarized in Table 1.

**Table 1:** Timeline of the episode of care.

Time point	Clinical event
19 months before admission	Severe blunt trauma caused intracranial hematoma, multiple cerebral contusions and lacerations, pelvic fracture, femoral fracture, and pulmonary contusion. The patient received surgical intensive care and orthopedic surgery.
Post-trauma interval	Intermittent dyspnea, chest tightness, postprandial fullness, abdominal distension, and abdominal pain developed without systematic reassessment.
Emergency admission	More than 20 h of nausea and vomiting with abdominal pain. Oxygen saturation was 94%, with no supplemental oxygen recorded. Examination identified diminished right breath sounds and bowel sounds in the lower right hemithorax.
Diagnostic assessment	Chest-abdominal CT showed right diaphragmatic discontinuity with herniation of the stomach, liver, gallbladder, and hepatic flexure into the right hemithorax.
Hospital day 3	After preoperative resuscitation and respiratory and metabolic optimization, transabdominal laparoscopy achieved adhesiolysis, reduction of viable organs, and mesh-free primary closure of an approximately 8.0 cm × 15.0 cm posterolateral defect with robust, approximable edges.
First postoperative week	Respiratory and gastrointestinal symptoms resolved. In-hospital CT showed restoration of the abdominal viscera without recurrent intrathoracic herniation on that study. No longer-term CT was available.

### 2.2 Clinical findings

On admission, temperature was 37.4 degrees C, pulse 85 beats/min, respiratory rate 20 breaths/min, blood pressure 127/91 mmHg, and peripheral oxygen saturation 94%. No supplemental oxygen was recorded, and no formal pulmonary function test result was available. The abdomen was soft, with upper abdominal tenderness but no rebound tenderness or guarding. Right-sided respiratory excursion was reduced. Breath sounds were diminished over the right lung and absent at the right base, while bowel sounds were audible in the lower right hemithorax. This combination of remote trauma, mixed respiratory and gastrointestinal symptoms, and intrathoracic bowel sounds raised concern for delayed TDH with visceral obstruction.

### 2.3 Diagnostic assessment

Chest-abdominal CT demonstrated focal discontinuity of the right hemidiaphragm. The stomach, liver, gallbladder, and hepatic flexure of the colon had migrated into the right thoracic cavity (Figure 1). Together, the clinical and imaging findings established delayed right-sided TDH presenting with acute obstructive gastrointestinal symptoms.

**Figure 1:**
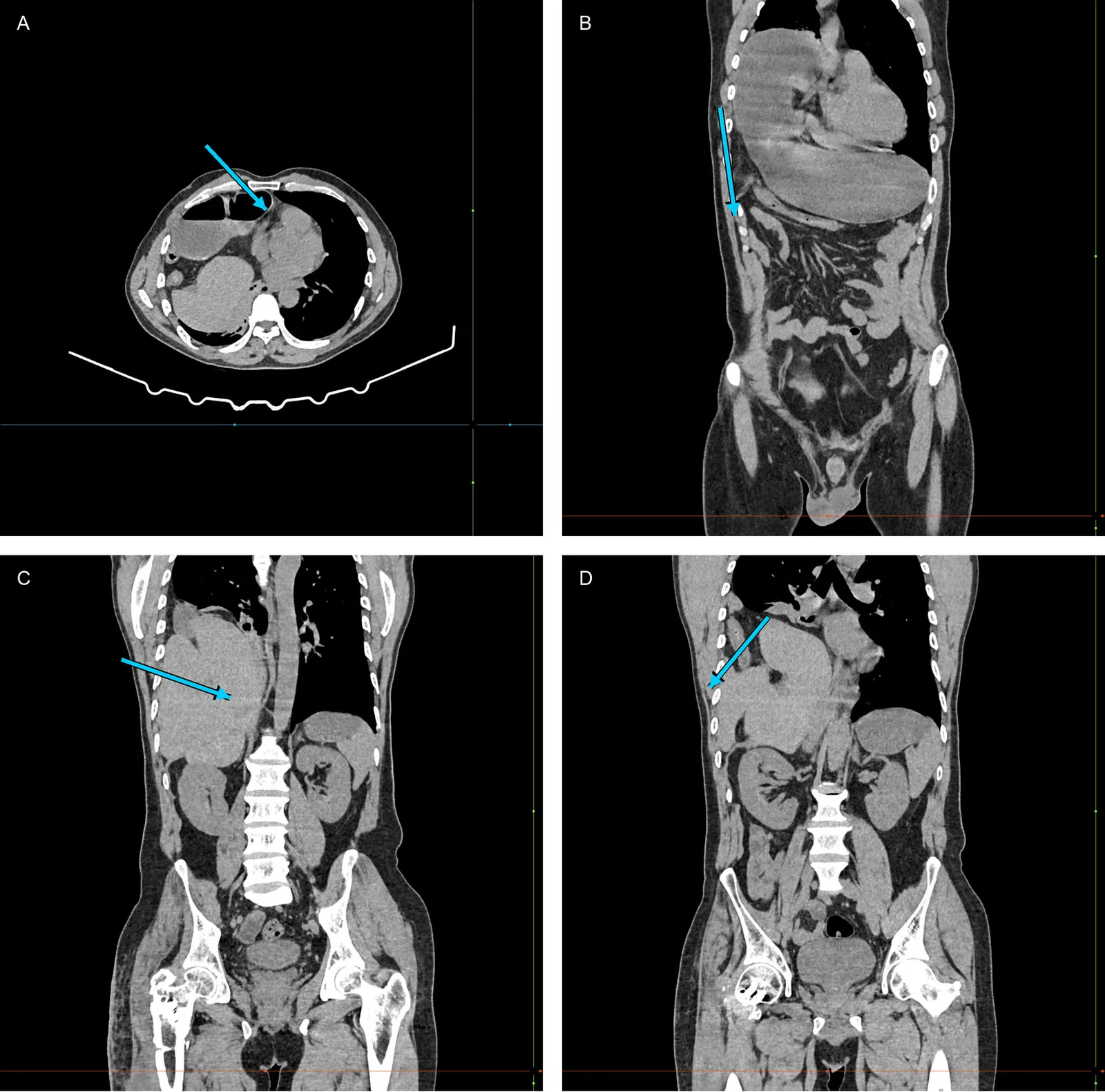
Preoperative chest-abdominal computed tomography. **(A)** An axial image shows gas-containing stomach or colon above the expected right hemidiaphragm (arrow). **(B)** A coronal image shows abdominal viscera entering the right hemithorax through the diaphragmatic defect (arrow). **(C)** A coronal image shows intrathoracic displacement of the liver (arrow). **(D)** A coronal image shows the posterolateral hernia and adjacent herniated viscera (arrow).

### 2.4 Therapeutic intervention

The patient remained hemodynamically stable, with no peritoneal signs or evidence of visceral perforation requiring immediate laparotomy. On hospital day 3 of the current admission, laparoscopic transabdominal exploration was performed. The brief preoperative interval allowed intravenous fluid resuscitation, correction of electrolyte and hematologic abnormalities, respiratory optimization, and assessment for associated thoracoabdominal injury. The liver, stomach, gallbladder, and hepatic flexure were herniated through a right posterolateral defect measuring approximately 8.0 cm × 15.0 cm. The right lung was compressed. Adhesions between the liver and diaphragm were divided with an ultrasonic device, and the herniated organs were gently reduced into the abdomen. The bowel remained viable, without ischemia or necrosis.

After adhesiolysis and circumferential mobilization, the diaphragmatic muscle and fascial edges were thick and robust, without marked atrophy, thinning, or friability. The edges could be approximated without a residual gap, visible tissue tearing, or excessive tension. The defect was repaired using multidirectional primary suture closure. Sequential bites were placed at different tissue depths and orientations to plicate the defect, distribute edge tension across several vectors, and restore a dome-like contour. A 2-0 barbed suture and a 1-0 suture were used. No mesh was placed because stable edge-to-edge closure was achieved and bridging reinforcement was unnecessary.

### 2.5 Follow-up and outcomes

Dyspnea, chest tightness, postprandial fullness, abdominal distension, and abdominal pain resolved after surgery. Postoperative CT showed restoration of the abdominal viscera below the diaphragm and re-expansion of the right lung. Mild residual right basilar atelectatic opacity was present, without recurrent intrathoracic herniation (Figure 2). The latest available radiographic follow-up was this in-hospital CT obtained within the first postoperative week. The available inpatient record did not document re-expansion pulmonary edema or another major postoperative complication. No longer-term CT was available, so durable recurrence-free status cannot be established.

**Figure 2:**
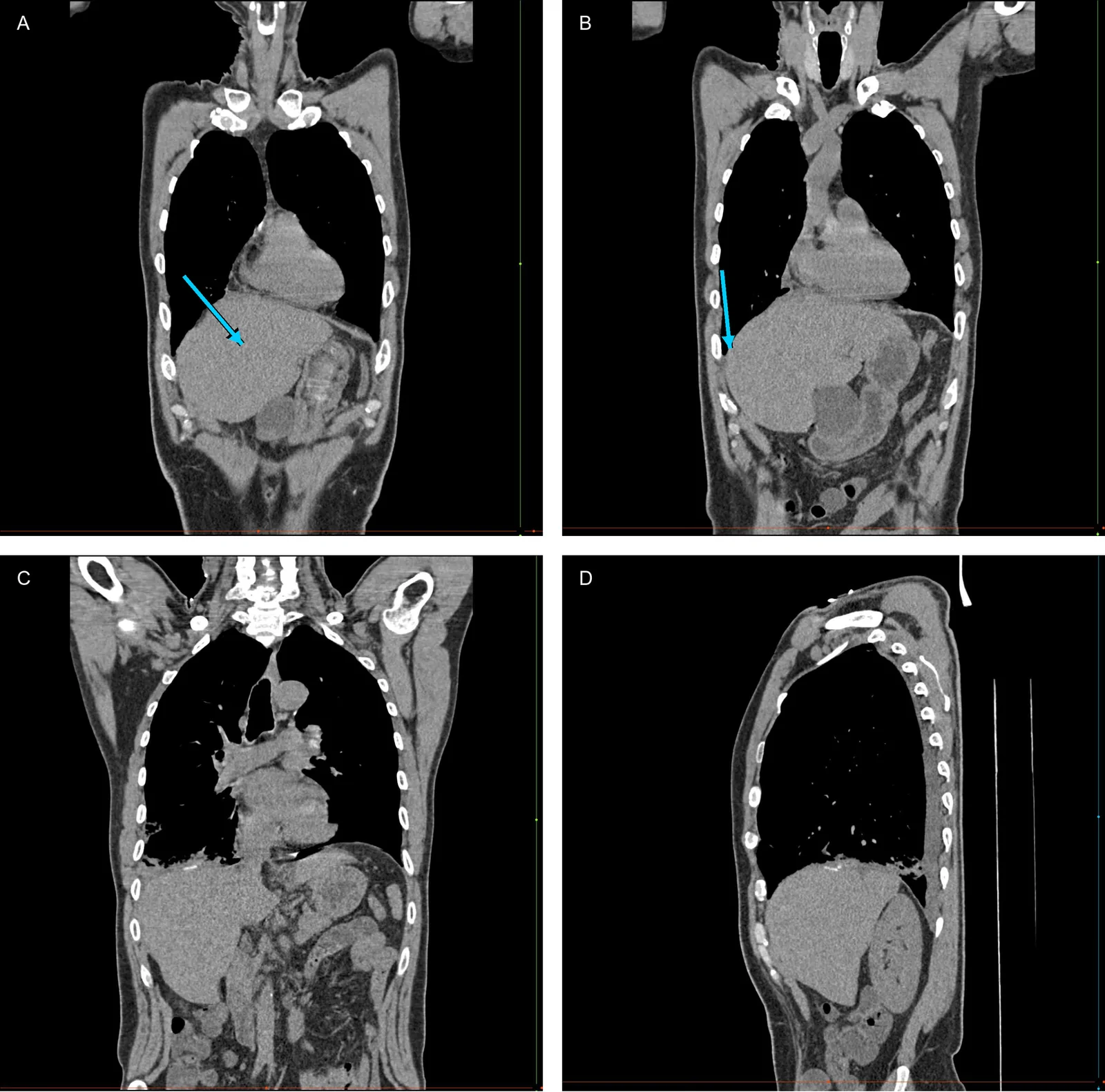
Postoperative chest-abdominal computed tomography. **(A,B)** Coronal images show the liver restored below the right hemidiaphragm and the reconstructed diaphragmatic contour (arrows). **(C,D)** Coronal and sagittal images show restored thoracoabdominal anatomy, right-lung re-expansion with mild residual right basilar atelectatic opacity, and no recurrent intrathoracic herniation on the in-hospital CT obtained within the first postoperative week.

## 3 Discussion

### 3.1 Diagnostic pitfalls and comparison with previous reports

This case was recognized during an acute gastrointestinal presentation, while the bowel was still viable. Three factors reinforced the diagnostic delay. Life-threatening injuries dominated the initial trauma admission, subsequent symptoms crossed respiratory and gastrointestinal systems, and the liver masked a right-sided defect. Intrathoracic bowel sounds finally connected the remote injury to the current presentation. Targeted CT review then confirmed both diaphragmatic discontinuity and organ migration (3–5).

The initial trauma CT could not be retrospectively reviewed because the original dataset and lung-window images were unavailable. We therefore cannot determine whether a subtle diaphragmatic sign was present but overlooked at that time. This distinction is important. The case identifies a missed clinical opportunity for structured reassessment after high-energy trauma, but it does not provide radiologic proof that a lesion was overlooked on the original scan.

The recent report by Yun et al. illustrates the same imaging blind spot from a different direction. Partial hepatic herniation was initially interpreted as biliary disease, and tension pneumothorax developed during attempted laparoscopic cholecystectomy. Re-review of serial CT images identified a hepatic collar sign (5). Together, these cases support deliberate inspection of the entire right hemidiaphragm when upper abdominal symptoms follow remote trauma.

A practical diagnostic sequence emerges from these observations. A history of high-energy thoracoabdominal trauma should first be linked to otherwise discordant respiratory and postprandial symptoms. Abnormal thoracic findings, particularly bowel sounds or markedly reduced basal breath sounds, should then trigger targeted multiplanar CT review. That review should define the defect, herniated organs, obstruction, and any sign of compromised perfusion rather than stopping at an elevated hemidiaphragm.

Recent clinical datasets show that CT limitations remain relevant. In a three-center series, only 14 of 25 CT-evaluated patients were diagnosed preoperatively, and associated injuries occurred in 92% of blunt-trauma cases (12). A 2026 trauma-registry study found a diagnostic delay exceeding 24 h in 8 of 22 injuries; 7 were missed despite CT (13). In a larger CT cohort, diaphragmatic discontinuity and intrathoracic herniation were the most sensitive signs of blunt injury, with sensitivities of 65%–77%. Overall reader sensitivity remained 76%–82% (14). Although the three-center study excluded chronic cases, these data support the same mechanism: competing injuries and imperfect CT detection can obscure a diaphragmatic tear. A structured, mechanism-aware review is therefore more defensible than reassurance from a nominally negative scan.

Young and Mukherjee reported strangulation of the right colon 18 months after blunt trauma. Emergency laparotomy, right colectomy, and ileocolostomy were required (6). Yadav et al. described delayed right-sided TDH with closed-loop ileal obstruction, perforation, and gangrene, treated by laparotomy and double-barrel ileostomy (7). Our patient presented after a similar delay, but his bowel remained viable. This timing permitted preservation of all herniated organs and a minimally invasive repair. Intermittent symptoms should therefore not be treated as reassurance after high-energy thoracoabdominal trauma.

### 3.2 Laparoscopic management and repair strategy

The transabdominal approach offered two advantages in this patient. It enabled direct assessment of the stomach and colon during an acute gastrointestinal presentation. It also allowed reduction and repair without repositioning. Laparoscopy should remain selective. Hemodynamic instability, threatened viscera requiring rapid resection, dense intrathoracic adhesions, poor visualization, or unsafe reduction should lower the threshold for thoracoscopy or open conversion (1, 9, 10).

Published right-sided repairs demonstrate why approach selection cannot be based on delay or defect size alone. Jain et al. used laparoscopic mesh reinforcement for a large hernia containing liver, gallbladder, small bowel, and omentum 29 years after penetrating trauma (8). Hashimoto et al. used combined thoracoscopy and laparoscopy for a 36-year delayed defect with extensive intrathoracic adhesions (9). Cornwell et al. repaired a seven-year delayed right-sided TDH laparoscopically after liver mobilization and two-layer primary closure (10). Rossetti et al. had already demonstrated laparoscopic mesh-free repair of a giant right post-traumatic defect in 2005 (15). That precedent shows that right-sided location and omission of mesh are not novel in isolation. The clinically relevant issue is how patients are selected and how a broad defect is closed while preserving viable viscera.

A large anatomic comparator was reported by Kesavaramanujam et al. Their 15 cm × 10 cm defect contained the entire liver, gallbladder, hepatic flexure, and part of the duodenum. Repair required a combined abdominal-thoracic strategy and mesh reinforcement (16). In contrast, adhesions in our patient were manageable from the abdomen. The mobilized muscle and fascial edges were robust and approximated without a residual gap, tissue tearing, or excessive tension. This permitted plication and mesh-free closure of the 8.0 cm × 15.0 cm defect. These contrasts suggest that adhesion distribution, organ viability, tissue quality, visualization, and achievable edge approximation may be more useful operative determinants than chronicity alone.

Retrospective series support laparoscopic repair in selected stable patients but also show that large defects usually receive reinforcement (17–19). A 23-patient series routinely reinforced defects measuring at least 5 cm and used a muscle-flap-plus-mesh reconstruction for defects measuring at least 10 cm (17). In a later 29-patient series, only 6 patients underwent simple suture repair; most laparoscopic repairs incorporated mesh, and 2 patients required open conversion (18). WSES guidance suggests mesh when direct suture closure cannot be achieved, although the supporting evidence is of low quality (1). Our case therefore represents a selected departure from common practice. The decision was based on robust tissue and stable approximation, not on defect size alone. The multidirectional plication was an intraoperative adaptation to distribute tension and should not be interpreted as evidence that mesh-free repair is superior for large defects. Because longer-term imaging was unavailable, the durability of this choice remains unproven.

Chronic compression can reduce ventilated right-lung volume, promote basal atelectasis, and impair ventilation-perfusion matching. These effects are consistent with this patient's dyspnea, reduced basal breath sounds, and admission oxygen saturation of 94%. Reduction of long-standing intrathoracic viscera may abruptly re-expand the compressed lung. Re-expansion pulmonary edema has been reported after repair of delayed post-traumatic diaphragmatic hernia (20, 21). This rare risk supports lung-protective ventilation, careful fluid management, and postoperative oxygenation and imaging surveillance. In this patient, early CT showed right-lung re-expansion with mild residual basal atelectatic opacity, and the available record did not document re-expansion pulmonary edema.

### 3.3 Strengths and limitations

The report is strengthened by a clear trauma-to-symptom timeline, a distinctive physical finding, direct operative confirmation, and paired preoperative and postoperative CT images. Its educational value lies in the diagnostic sequence and operative decision process rather than in a claim of rarity.

Several limitations constrain interpretation. This single case cannot compare abdominal, thoracic, combined, or open approaches. Tissue quality and residual closure tension were assessed qualitatively rather than by a standardized or biomechanical method. The original trauma CT and pulmonary function tests were unavailable, preventing retrospective assessment of an early diaphragmatic sign or quantitative respiratory impairment. The in-hospital follow-up CT establishes early anatomic restoration but not the long-term durability of primary closure for a large defect.

## 4 Conclusion

Delayed right-sided TDH should be considered when respiratory and meal-related gastrointestinal symptoms persist after high-energy blunt trauma. Bowel sounds in the right hemithorax should trigger deliberate multiplanar CT review before strangulation occurs. In stable patients with viable viscera and manageable adhesions, transabdominal laparoscopy can provide reduction, organ assessment, and repair in one procedure. The approach and need for mesh should be individualized according to adhesions, tissue quality, and achievable edge approximation.

## 5 Patient perspective

The patient reported that the breathing difficulty, chest tightness, postprandial fullness, abdominal distension, and abdominal pain that had persisted after the trauma resolved after surgery.

## Data Availability

The raw data supporting the conclusions of this article will be made available by the authors, without undue reservation.
