# Supplementary material for: Case Report: Diagnostic pitfalls and laparoscopic repair of a delayed large right-sided traumatic diaphragmatic hernia
Source: Front Surg. 2026 Sep 22;13:1956256. doi: 10.3389/fsurg.2026.1956256 (PMC13638662; doi:10.3389/fsurg.2026.1956256)
Supplement: Supplementary file 1 [file Table1.docx]

**CARE 2013 Checklist**

Completed supplementary reporting checklist

| **Topic** | **Item** | **Reported in manuscript** |
| --- | --- | --- |
| Title | 1 | Title identifies the report as a Case Report. |
| Key words | 2 | Keywords (seven terms, including case report). |
| Abstract | 3a | Abstract - Background. |
| Abstract | 3b | Abstract - Case description: symptoms and clinical findings. |
| Abstract | 3c | Abstract - Case description: diagnosis, intervention, and outcome. |
| Abstract | 3d | Abstract - Conclusion. |
| Introduction | 4 | Introduction. |
| Patient information | 5a | Case description - Patient information. |
| Patient information | 5b | Case description - Patient information and clinical findings. |
| Patient information | 5c | Relevant medical history is reported; family, genetic, and psychosocial history was not applicable to the clinical focus. |
| Patient information | 5d | Case description - Patient information; Table 1. |
| Clinical findings | 6 | Case description - Clinical findings. |
| Timeline | 7 | Table 1. |
| Diagnostic assessment | 8a | Case description - Diagnostic assessment; Figure 1. |
| Diagnostic assessment | 8b | Introduction and Discussion - Diagnostic pitfalls and comparison with previous reports. |
| Diagnostic assessment | 8c | Case description - Diagnostic assessment. |
| Diagnostic assessment | 8d | Not applicable; prognostic staging was not relevant. |
| Therapeutic intervention | 9a | Case description - Therapeutic intervention. |
| Therapeutic intervention | 9b | Case description - Therapeutic intervention. |
| Therapeutic intervention | 9c | No change in the planned intervention was reported. |
| Follow-up and outcomes | 10a | Follow-up and outcomes; Patient perspective. |
| Follow-up and outcomes | 10b | Follow-up and outcomes; Figure 2. |
| Follow-up and outcomes | 10c | Outcome and symptom resolution are reported; adherence was not separately assessed. |
| Follow-up and outcomes | 10d | No adverse or unanticipated events were documented in the available record. |
| Discussion | 11a | Discussion - Strengths and limitations. |
| Discussion | 11b | Discussion - literature comparison and management strategy. |
| Discussion | 11c | Discussion and Conclusion. |
| Discussion | 11d | Conclusion. |
| Patient perspective | 12 | Patient perspective. |
| Informed consent | 13 | Ethics statement. |

Checklist source: Gagnier et al., J Med Case Rep. (2013) 7:223; https://www.care-statement.org/checklist
